# Mycorrhization of transgenic apple trees with increased resistance against fungal pathogens

**DOI:** 10.1186/1753-6561-5-S7-O55

**Published:** 2011-09-13

**Authors:** Tina Schäfer, Henryk Flachowsky, Stephan König, Stefan Hempel, Tesfaye Wubet, Andreas Peil, Michael Kaldorf, Magda-Viola Hanke, François Buscot

**Affiliations:** 1 UFZ-Helmholtz Centre for Environmental Research, Department of Soil Ecology, Halle, Germany & University Leipzig, Faculty of Biological Science, Pharmacy and Psychology, Institute of Biology I, Department of Terrestrial Ecology, Leipzig, Germany; 2 Julius Kühn-Institute, Federal Research Centre for Cultivated Plants, Institute for Breeding Research on Horticultural and Fruit Crops, Dresden, Germany; 3 UFZ-Helmholtz Centre for Environmental Research, Department of Soil Ecology, Halle, Germany; 4 Freie Universität Berlin, Institute of Biology, Dahlem Center of Plant Sciences, Department of Plant Ecology, Berlin, Germany; 5 Gesellschaft für Umwelt- und Innenraumanalytik mbH, Mönchengladbach, Germany & University Leipzig, Faculty of Biological Science, Pharmacy and Psychology, Institute of Biology I, Department of Terrestrial Ecology, Leipzig, Germany

## 

The project 'Mycorrhizal symbioses of transgenic apple trees with increased resistance against fungal pathogens' (Subproject of the framework 'Biosafety of transgenic woody plants', supported by the German Federal Ministry for Education and Research) investigates whether the transformation of apple trees with fungal chitinase encoding genes - besides increasing resistance against fungal pathogens - also interferes with mycorrhizal fungi. Pathogenic fungi such as the ascomycete species *Venturia inaequalis*, the pathogenic agent of apple scab, cause severe damage in apple cultivation. In commercial apple production, pesticides are employed frequently and in high quantities. Alternative methods such as the use of mycoparasitic *Trichoderma* fungi to control fungal plant pathogens have been explored [1]. Genetic engineering of apple cultivars with *Trichoderma* chitinases has lead to an increase of the trees’ resistance against apple scab [2]. However, like many fruit tree species, apple trees form symbiotic associations with arbuscular mycorrhizal fungi (AMF) at their roots. AMF receive fixed carbon compounds, while the plants benefit from the symbiosis by enhanced uptake of nutrients and water, promoted growth, and increased resistance against biotic and abiotic stress [3]. Like in most higher fungi, the cell walls of AMF contain chitin as a major skeletal component [4]. Consequently, the symbiosis between apple and AMF may be negatively affected by the expression of *Trichoderma* chitinase genes within the plant. At the Julius Kühn-Institute in Dresden, the apple cultivar 'Pinova' was transformed by introducing the construct pBIN (Endo + Nag) into the transgenic lines. With the help of promoter CaMV 35S the transgenic lines constitutively express endochitinase gene *ech*42 and exochitinase gene *nag*70 from the biocontrol fungus *Trichoderma atroviride.* In order to study whether the constitutive expression of *ech*42 and *nag*70 has an effect on mycorrhization of plants with transgenic roots (local effect) and – as apple cultivars are propagated vegetatively by grafting– whether expression of *ech*42 and *nag*70 in a transgenic scion grafted on a non-transgenic rootstock has an effect on mycorrhization (systemic effect), a series of greenhouse experiments was conducted. In a first experiment, non-grafted clones of wildtype apple cultivar ‘Pinova’ and two transgenic lines were cultivated in artificial substrate and inoculated with two AMF species, *Glomus intraradices* and *G. mosseae*. After four months of cultivation, root colonization rates were significantly lower in the transgenic lines compared to the non-transformed cultivar 'Pinova' [5]. Samples were taken again after another four months of cultivation under enhanced inoculation pressure (increased inoculum mass, decreased nutrient availability in the substrate). The experimental approach was broadened by using soil from different field sites in order to supply the sample trees with complex soil matter, AMF adapted to the very soil conditions, and the appropriate soil bacteria which also play a role in the formation of mycorrhizal associations. Two sets of non-grafted sample trees (clones of apple cultivar 'Pinova' and two transgenic lines) were cultivated in the greenhouse; one set in pots with soil from an intensively managed apple plantation, the other set in pots with soil from an extensively managed apple orchard. Root sampling was conducted three and six months after transferring the plants to soil. To assess the systemic effect (expression of *ech*42 and *nag*70 in a transgenic scion grafted on a non-transgenic rootstock) on mycorrhization, two further experiments were carried out. Grafted sample trees (clones of apple cultivar 'Pinova' and two transgenic lines, all grafted on commonly grown rootstock M9) were cultivated in the greenhouse in pots with artificial substrate and inoculum of *Glomus intraradices* and *G. mosseae*. Root samples were taken after four and eight months. A second set of grafted sample trees was cultivated in pots with soil from an intensively managed plantation in the ‘semi-natural’ environment of a gossamer cage. Root samples were taken after 12 and 24 months. For all experiments, root colonisation rates were determined microscopically. Molecular methods – PCR with taxa specific primer sets [6], cloning of PCR products, Sanger sequencing – were applied to study the impact of the transformation on the composition of the fungal communities associated with the roots of the sample trees cultivated in soil. Chitinase activity in leaves and roots of sample trees was determined using fluorescence spectrometry and substrates 4-methylumbelliferyl N-acetyl-β-D-glucosaminide for exochitinase and 4-methylumbelliferyl β-D-N,N′,N′′-triacetylchitotrioside for endochitinase activity. The results of this biosafety research concerned with potential adverse effects on benefitial soil fungi will be presented and discussed.
